# Amaryllidaceae Alkaloids and Phenolic Acids Identification in *Leucojum aestivum* L. Plant Cultures Exposed to Different Temperature Conditions

**DOI:** 10.3390/molecules31020258

**Published:** 2026-01-12

**Authors:** Agata Ptak, Marzena Warchoł, Emilia Morańska, Dominique Laurain-Mattar, Rosella Spina, François Dupire, Piotr Waligórski, Magdalena Simlat

**Affiliations:** 1 Department of Plant Breeding, Physiology and Seed Science, University of Agriculture in Krakow, 31-140 Krakow, Poland; magdalena.simlat@urk.edu.pl; 2 The Franciszek Górski Institute of Plant Physiology, Polish Academy of Sciences, 30-239 Krakow, Poland; m.warchol@ifr-pan.edu.pl (M.W.); p.waligorski@ifr-pan.edu.pl (P.W.); 3 Department of Plant Biology and Biotechnology, University of Agriculture in Krakow, 31-425 Krakow, Poland; emilia.moranska@urk.edu.pl; 4 Université de Lorraine, INRAE, LAE, F-54000 Nancy, France; dominique.mattar@univ-lorraine.fr (D.L.-M.); rosella.spina@univ-lorraine.fr (R.S.); 5 Université de Lorraine, CNRS, L2CM, F-54000 Nancy, France; francois.dupire@univ-lorraine.fr

**Keywords:** galanthamine, lycorine, phenolic acids, gene expression, *Leucojum aestivum* plants, abiotic stress, in vitro

## Abstract

Amaryllidaceae alkaloids are of notable pharmacological relevance. For instance, galanthamine is used in the treatment of Alzheimer’s disease, while other alkaloids (lycorine, crinine, etc.) derived from Amaryllidaceae plants are also of great interest because they exhibit antitumour, antiviral, antibacterial, antifungal, antimalarial, analgesic and cytotoxic properties. Phenolic acids comprise a group of natural bioactive substances that have commercial value in the cosmetic, food and medicinal industries due to their antioxidant, anticancer, anti-inflammatory and cardioprotective potential. In the present study, the effect of temperature (15, 20, 25 and 30 °C) on Amaryllidaceae alkaloid and phenolic acid biosynthesis in *Leucojum aestivum* in vitro plant cultures was investigated. The highest diversity of alkaloids (i.e., galanthamine, crinan-3-ol, demethylmaritidine, crinine, 11-hydroxyvitattine, lycorine, epiisohaemanthamine, chlidanthine) was noted in plants cultured at 30 °C. By contrast, ismine and tazettine were only present in plants cultured at 15 °C. Temperatures of 20 °C and 30 °C were found to stimulate galanthamine accumulation. The highest lycorine content was noted in plants grown at temperatures of 15 and 30 °C, and it was negatively correlated with the expression of the gene that encodes the cytochrome P450 96T (CYP96T) enzyme which catalyses a key step in the biosynthesis of different types of Amaryllidaceae alkaloids. This observation may reflect temperature-induced shifts in metabolic flux among different branches of Amaryllidaceae alkaloid biosynthesis. The observed stimulating effect of a 15 °C temperature on the chlorogenic, caffeic, *p*-coumaric, sinapic, ferulic and isoferulic acid content was in line with the highest expression of a gene that encodes the tyrosine decarboxylase (TYDC) enzyme, which is involved in plant stress response mechanisms. At 30 °C, however, the highest content of the caffeic, vanillic, *p*-coumaric and isoferulic acids was noted.

## 1. Introduction

Amaryllidaceae alkaloids are distinctive chemical markers belonging to the family Amaryllidaceae. The exploration of Amaryllidaceae alkaloids commenced in 1877 with the isolation of lycorine from *Narcissus pseudonarcissus* [1]. Subsequently, interest in these alkaloids surged due to their broad spectrum of bioactivities. A pivotal breakthrough came with the isolation of galanthamine from *Galanthus woronowii* in 1952 by Proskurina and Yakovleva, who revealed its long-lasting, selective, reversible and competitive inhibition of the enzyme acetylcholinesterase. Moreover, since 2000, galanthamine has been registered as a drug for treating Alzheimer’s disease [2,3,4]. Lycorine is currently undergoing clinical trials concerning its significant anticancer activities against various types of cancer [5,6]. In addition, lycorine has shown promise in combatting SARS-CoV-2 infection due to its antiviral activity [7]. Indeed, many of the Amaryllidaceae alkaloids exhibit potent biological and pharmacological properties, including antitumour, antiviral, antibacterial, antifungal, antimalarial, analgesic and cytotoxic properties [4,8,9,10,11].

Amaryllidaceae alkaloids also have significant value in terms of agriculture. For instance, recent studies indicated that lycorine displays potent activities against various agricultural pests (e.g., *Plutella xylostella*, *Aphis citricola*, tobacco mosaic virus, *Phytophthora capsici*, *Botrytis cinerea*, *Fusarium graminearum*, *Magnaporthe oryzae*), which suggests its potential as a candidate biopesticide [12]. To date, over 650 Amaryllidaceae alkaloids have been described, and their chemical repertoire is associated with ongoing expansion [13]. However, the use of Amaryllidaceae alkaloids in both the pharmaceutical industry and agriculture remains difficult due to their limited commercial availability.

Galanthamine is a major pharmacologically relevant alkaloid of the Amaryllidaceae family [2]. Currently, for pharmaceutical applications, it is obtained by means of chemical synthesis or extraction from plants. The industrial-scale chemical synthesis process for galanthamine was first developed in 1998 [14]. Yet, as galanthamine contains three asymmetric carbons, its chemical production is time-consuming and costly owing to its structural complexity and the challenges in relation to maintaining its configuration during synthesis [15,16]. Nevertheless, industrial scale synthesis remains limited, and plant extraction continues to be the primary commercial source. Hence, obtaining this alkaloid on an industrial scale via plant extraction has been pursued since the 1960s when the Bulgarian company Sopharma began producing the drug Nivalin^®^ (Sopharma AD, Sofia, Bulgaria) by extracting galanthamine from naturally growing *Leucojum aestivum*, although excessive harvesting of this plant resulted in it being protected [17]. Overharvesting led to strong declines in natural populations, prompting legal protection in several regions. Hence, galanthamine for pharmaceutical application is currently sourced from cultivated plants like *Narcissus* sp., *L*. *aestivum*, *Lycoris radiata* and *Ungernia victoris*, with galanthamine levels ranging from 0.1% to 0.52% depending on the species and cultivation conditions [18].

These limitations have stimulated research into alternative production methods, including plant cell cultures, elicitation strategies and metabolic engineering approaches. Review papers by Laurain-Mattar and Ptak [18], Georgiev et al. [19], Kaur et al. [20] and Koirala et al. [21] all investigated plant biotechnology employing in vitro systems. In this regard, the initial articles on the biosynthesis of galanthamine and lycorine in *L*. *aestivum* in vitro culture were published during the early 1990s and concerned shoot cultures [19]. Subsequent research focused on the possibility of Amaryllidaceae alkaloids biosynthesis via embryogenic callus, somatic embryos and plant cultures of *L*. *aestivum* [18]. However, as embryogenic callus were characterised by the lowest ability to biosynthesise alkaloids, such work mainly focused on the potential of somatic embryos and the plants obtained from them in relation to the production of Amaryllidaceae alkaloids. Various elicitors were used to stimulate the biosynthesis of alkaloids in these cultures—namely, methyl jasmonate, salicylic acid, 1-aminocyclopropane-1-carboxylic acid, 2-chloroethylphosphonic acid [22], melatonin, sodium chloride [23], carbohydrates [24], autoclaved endophytic bacteria [25] and light-emitting diode (LED) lights in somatic embryos and plant cultures [26]. Still, to render in vitro production of Amaryllidaceae alkaloids economically viable, it is necessary to utilise various biotechnological approaches and identify the most efficient elicitor of the biosynthesis of specialized metabolites [20].

Phenolic acids are usually produced as a defence mechanism in response to an attack on the plant tissue or in a stressful environment. Indeed, under abiotic and biotic stress conditions, phenolic acid biosynthesis is usually increased when compared with that under normal conditions [27]. Phenolic acids represent an extensively studied group of phenolic compounds due to their important biological properties, particularly their strong antioxidant activity. Additionally, studies have shown the protective role of phenolic acids in degenerative diseases such as cardiovascular disease, cancer, diabetes and inflammation [28]. The technological and functional properties of phenolic acids also render them interesting compounds for use in the pharmaceutical, food and cosmetics industries. To meet the growing research and industrial demands, it is essential to achieve the biosynthesis of these compounds in vitro. Hence, numerous reports have been directed towards the production of phenolic acids via in vitro plant cultures [29,30]. However, little research has been performed on the biosynthesis of phenolic acids using in vitro cultures of Amaryllidaceae plants. Only Morańska et al. [26] demonstrated the presence of the chlorogenic, *p*-hydroxybenzoic, caffeic, syringic, *p*-coumaric, ferulic, sinapic and benzoic acids in *L*. *aestivum* in vitro plants grown under different light conditions. It must also be emphasised that information on the content of individual phenolic acids in naturally growing Amaryllidaceae plants is similarly very limited. For example, Nikolova and Gevrenova [31] identified seven phenolic acids in extracts derived from *Sternbergia colchiciflora*, *Galanthus nivalis*, *Galantus elwesii*, *L*. *aestivum* and *Pancratium maritimum* grown in Bulgaria. Moreover, a few studies have been conducted to determine the total content of phenolic compounds in *L*. *aestivum* plants [32,33,34,35].

Temperature is a major factor influencing the in vitro growth and development of plants. Plants grow better and produce higher biomass yields under conditions of lower stress. In fact, when exposed to extreme temperatures, in vitro cultures increase the production of specialized metabolites to protect against such stressful conditions [36]. In this regard, Ivanov et al. [37,38] showed that the Amaryllidaceae alkaloid profile of *L*. *aestivum* in vitro shoots cultivated in the Rita^®^ bioreactor (Vitropic, St-Mathieu de Tréviers, France) may change depending on the applied temperature. Furthermore, it is common knowledge that both low and high temperature—as an abiotic stress factor—causes the accumulation of reactive oxygen species (ROS) and, therefore, negatively alters the growth of plants. To mitigate the effects of ROS formation, plants have evolved antioxidant systems based on the production of low-molecular-mass antioxidants and/or the action of antioxidant enzymes, for example, superoxide dismutase (SOD), catalase (CAT) and peroxidase (POD) [39]. To date, no study concerning the impact of temperature on Amaryllidaceae alkaloid biosynthesis has been carried out in *L*. *aestivum* plant cultures. Hence, while it is known that the biosynthesis of Amaryllidaceae alkaloids depends on the degree of tissue differentiation and may also occur differently depending on the type of in vitro culture [18], the effect of temperature on phenolic acid biosynthesis in in vitro cultures of Amaryllidaceae plants has not yet been studied.

To address this gap in the literature, the present study sought to evaluate the response of *L*. *aestivum* in vitro plant cultures (e.g., plant growth, photosynthetic pigment and soluble sugar content, activities of antioxidant enzymes, Amaryllidaceae alkaloid and phenolic acid biosynthesis) to temperature stress conditions. Moreover, for the first time, the expression of genes involved in different steps of the Amaryllidaceae alkaloid biosynthetic pathway was analysed under different temperature conditions to gain deeper insight into the molecular mechanism of enhanced alkaloid biosynthesis in *L*. *aestivum*.

## 2. Results and Discussion

### 2.1. Effect of Temperature on In Vitro Plant Growth

**Figure 1:**
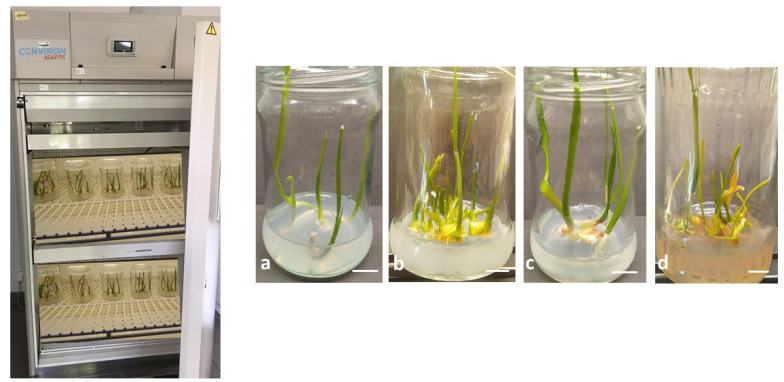
Visual appearance of *L*. *aestivum* plants after 28 days of in vitro cultivation in temperature: (**a**) 15 °C, (**b**) 20 °C, (**c**) 25 °C, (**d**) 30 °C; Scale bars: 1 cm (**a**,**c**), 0.8 cm (**b**,**d**).

*Leucojum aestivum* plants were grown at temperatures of 15, 20, 25 and 30 °C for 4 weeks. It was observed that the temperature significantly affected the morphology and development of the plants (Figure 1). More specifically, a temperature of 25 °C favoured leaf formation (2.68 leaves per plant). By contrast, fewest leaves were induced on plants grown at temperatures of 15 °C and 30 °C (an average of 2.16 times fewer than at 25 °C) (Table 1). All of the leaves were green, except for the leaves of the plants grown at 30 °C, which tended to turn brown in the second week of growth (Figure 1d).

Regarding root formation, the temperature had a marked influence on related processes. Here, the plants produced on average of 2.58 times more new roots at 15 °C and 25 °C than at 20 °C, while a temperature of 30 °C inhibited root formation (Table 1). Additionally, in the plants grown at 25 °C, both thickening of the shoot base and formation of bulbs were observed (Figure 1c).

The highest increase in the fresh weight (FW) of the *L*. *aestivum* plants’ biomass was recorded when the plants were cultured at 25 °C (430 mg). These plants also accumulated the greatest amount of dry weight (DW) (43.19%). The lowest fresh biomass increase was observed in relation to the plants grown at 15 °C (60 mg). In this case, the lowest DW accumulation was also recorded (24.94%), although this result did not differ statistically from those obtained when temperatures of 20 and 30 °C were applied (Table 1).

A temperature range of 17–25 °C is typically used to maintain in vitro cultures, although individual plant species may show optimal growth under different temperature regimes [36]. To date, research in *L*. *aestivum* plant cultures has only been carried out at a temperature of 25 °C [22]. For instance, Georgiev et al. [40] tested different temperature conditions (18, 22 and 26 °C) in terms of shoot cultures of *L*. *aestivum*. Similar studies have not been conducted in other plants from the Amaryllidaceae family obtained via the process of somatic embryogenesis. It is only known that *Leucojum vernum* [41] and *Narcissus confusus* [42] plants have been cultured at 25 °C. However, the conversion of *Narcissus* ‘Carlton’ somatic embryos into plants was performed at 20 °C [43], while somatic embryos of *Sternbergia lutea* were grown at 22 °C during the day and 18 °C during the night [44].

The present study demonstrated the unfavourable effect of extreme temperatures on *L*. *aestivum* plants’ development (15 °C and 30 °C inhibited leaf formation, while 30 °C inhibited root induction). According to Parolo et al. [45], the optimal temperature for many physiological processes, such as seed germination and the induction and differentiation of floral organs, in *L*. *aestivum* plants grown under natural conditions is 20–25 °C. Similarly, Shao et al. [46] reported that *Hippeastrum* spp. bulbs grown in soil at 25 °C were not only significantly larger in diameter but also exhibited greater FW and DW when compared with those grown at 30 °C.

Our study demonstrated the positive effect of a temperature of 25 °C on the FW increments and DW accumulation of *L*. *aestivum* plant cultures. Conversely, a shoot culture of *L*. *aestivum* cultivated in a glass-column bioreactor at 22 °C produced the highest amounts of dry biomass, while a temperature of 26 °C had a negative impact on the dry biomass content of the shoots [40]. This proves that each type of in vitro culture has different optimal conditions when it comes to cultivation.

### 2.2. Effect of Temperature on Photosynthetic Pigment Content

Temperature is one of the major environmental factors that can inhibit photosynthetic pigment biosynthesis and, therefore, affect photosynthesis [47].

In the analysed leaves of *L*. *aestivum* plants, when grown in different temperature conditions, no statistically significant differences were observed in terms of the pigment content (i.e., chlorophyll *a*, chlorophyll *b* and carotenoids). However, in the plants grown at 20 °C, the highest content of chlorophyll *a* and *b* was noted (44.33 and 27.29 µg/g FW, respectively). By contrast, the content of total carotenoids decreased with an increasing cultivation temperature (Table 2). A similar effect was observed by Georgiev et al. [40], albeit in shoot cultures of *L*. *aestivum*, where the accumulation of chlorophyll *a* and *b* in the tissue did not differ at temperatures of 18, 22 and 26 °C. According to the literature, a high temperature can reduce or inhibit carotenoid biosynthesis in plants grown in both in vitro and natural conditions [48,49]. Yet the effect of temperature on carotenoid biosynthesis in in vitro Amaryllidaceae plants has not previously been studied. Among other species, for example, *Lavandula viridis* and *Thymus transcaucasicus* which are Mediterranean plants from the Lamiaceae family, a temperature of 20 °C stimulated the biosynthesis of both carotenoids and chlorophylls [50]. In the case of *Nardostachys jatamansin*, an alpine plant (Caprifoliaceae), the photosynthetic pigment concentration was higher in shoots maintained at 13 °C than in those maintained at 24 °C [51]. There are clearly optimal temperature limits for each plant species, and exceeding the relevant range may affect a species’ physiological and biochemical functions. Additionally, under in vitro conditions, the reaction of plants also depends on the development phase of the cultures and the chemical factors used, including the composition of the medium [52].

### 2.3. Effect of Temperature on Soluble Sugar Content

The levels of soluble sugars in leaves, their transport and their storage are strictly regulated and influenced by the plant developmental stage, physiological activity and environmental conditions (e.g., CO_2_, light, temperature) [53]. In plant tissues, changes in the concentration of sugars occur continuously during the different developmental stages and serve as a signal of the availability of the products of photosynthesis in leaves [54].

**Figure 2:**
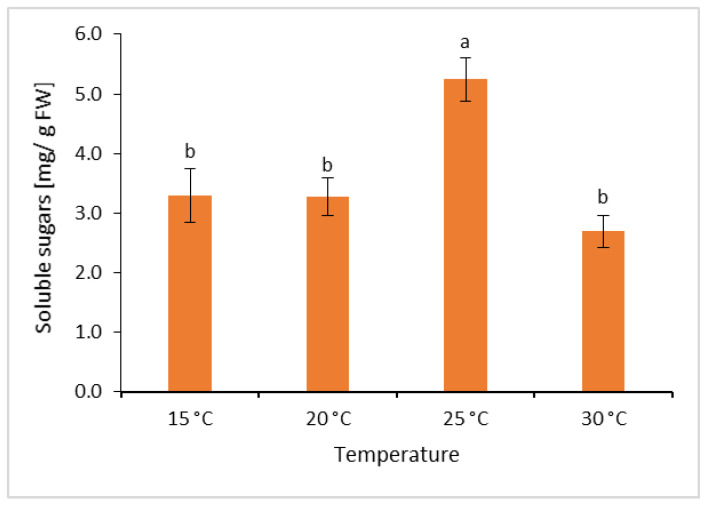
Effect of temperature on soluble sugars content in the leaves of *L*. *aestivum*. Bars represent mean values (*n* = 5) ± SE. Different letters indicate significant differences between means (Duncan’s multiple range test; *p* ≤ 0.05).

In this study, the highest content of soluble sugars—namely, 5.2 mg/g FW—was recorded in the *L*. *aestivum* plants growth at 25 °C, while in the plants grown at 15, 20 and 30 °C, the content of soluble sugars reached a similar level (3.3 mg/g FW, 3.3 mg/g FW and 2.7 mg/g FW, respectively) (Figure 2).

Lunn [55] emphasised that carbohydrate transport from the leaves via the phloem not only supplies the rest of the plant with carbon and energy required for growth but is also crucial for the formation and development of bulbs by means of storage product synthesis. This corresponds to the results of the present research, where the plants grown at 25 °C were characterised by the highest fresh and dry mass, and these plants also began to form bulbs. Increased soluble sugar accumulation during the vegetative stage of *Hippeastrum hybridum* ‘Red lion’ bulbs was reported by Zhang et al. [56]. They tested different temperatures and determined that 25 °C was not only more suitable than 30 °C or 20 °C for plant growth in this species but also that the sugar content in the bulbs grown at 25 °C rapidly increased during the experiment. Hence, research indicates there to be a relationship between carbohydrate metabolism and bulb development in, for example, *Lilium davidii* var. *unicolor*, *Hippeastrum vittatum* ‘Red lion’ and *Lilium japonicum* [56,57,58].

### 2.4. Effect of Temperature on Antioxidant Enzyme Activity

Abiotic stresses, including temperature (both heat and cold), in plants cause oxidative stress that arises from the formation imbalance between the production and inactivation of ROS. Overproduction of ROS can alter cellular homeostasis by adversely affecting plants’ physiological and biochemical processes [39]. To limit the negative effects of the presence of ROS in cells, plants have evolved enzymatic antioxidant systems wherein SOD, CAT and POD play key roles.

**Figure 3:**
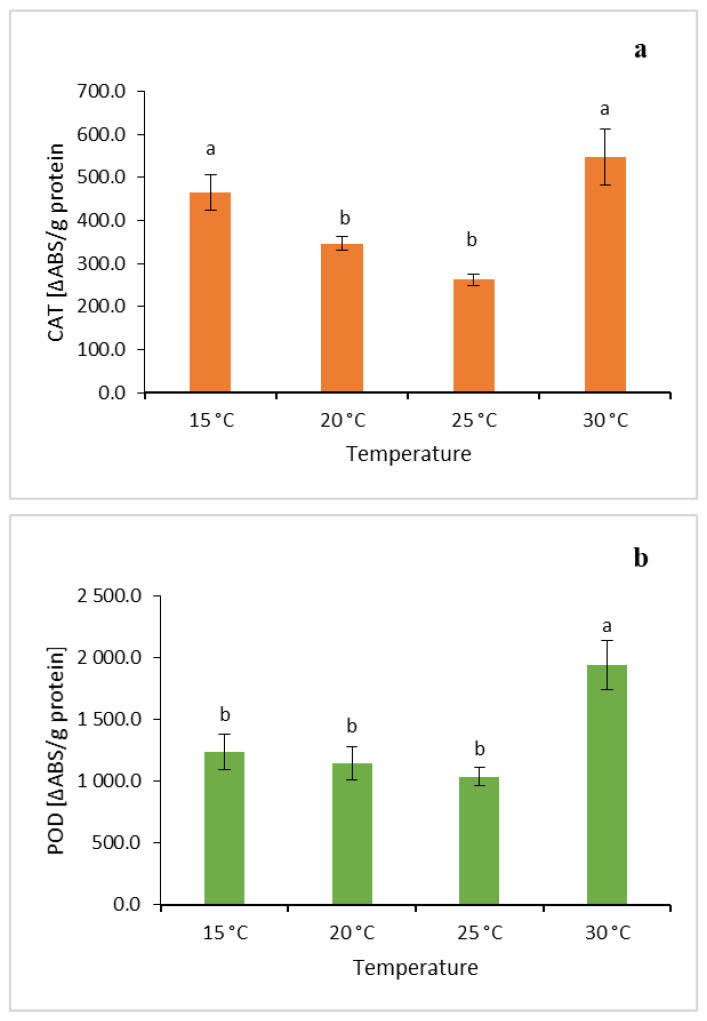

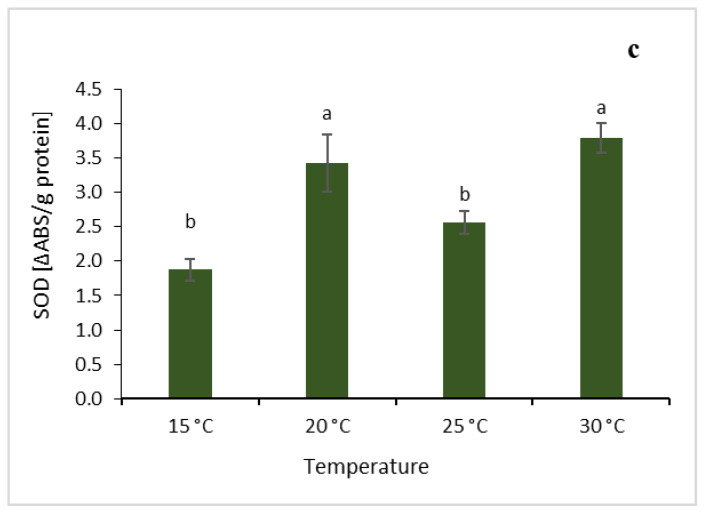
Effect of temperature on (**a**) catalase (CAT), (**b**) peroxidase (POD) and (**c**) superoxide dismutase (SOD) activities in the leaves of *L*. *aestivum*. Bars represent mean values (*n* = 5) ± SE. Different letters indicate significant differences between means (Duncan’s multiple range test; *p* ≤ 0.05).

In this study, the temperature during the growth of *L*. *aestivum* in vitro plants had a significant effect on the antioxidant enzyme activity (Figure 3). The highest activity of the CAT enzyme was observed when the plants were grown at 15 °C and 30 °C (464.36 ΔABS/g protein and 546.50 ΔABS/g protein, respectively) (Figure 3a). The activity of the POD enzyme was also the highest at 30 °C, amounting to 1939.47 ΔABS/g protein (Figure 3b).

Enzymatic reactions of plants to temperature stress were reported by Shohael et al. [59] in somatic embryos of *Eleutherococcus senticosus*, where a low temperature (12 °C) caused a significant increase in CAT activity, similar to the findings of this study. In the case of SOD, the highest enzyme activity was observed at 20 °C (3.43 ΔABS/g protein) and 30 °C (3.79 ΔABS/g protein), contrary to the findings reported by Shohael et al. [59], where a temperature of 30 °C decreased the enzymatic activity of SOD. The lowest enzymatic activities of CAT (262.75 ΔABS/g protein) and POD (1037.13 ΔABS/g protein) were noted at 25 °C, while the lowest activity of SOD (1.88 ΔABS/g protein) was observed at 15 °C (Figure 3c). While studies conducted by Abarca et al. [60] and Samis et al. [61] have showed increased induction of SOD in response to low temperatures, in this study the lowest SOD activity was noted in the plants grown at 15 °C.

Meanwhile, the present results clearly demonstrated that the highest activity of all three enzymes was obtained when the plants were grown at 30 °C. The negative effect of a 30 °C temperature on the *L. aestivum* plants’ growth (i.e., the number of leaves, the number of roots, the tendency towards browning and death of plants) and the high antioxidant enzyme activities confirm the sensitivity of *L. aestivum* plants to high temperatures and the induction of an oxidative stress response under such conditions. However, it is known that the optimal temperature for many physiological processes in *Leucojum aestivum* plants grown under natural conditions is 20–25 °C [45].

### 2.5. Effect of Temperature on Phenolic Acids Biosynthesis

**Figure 4:**
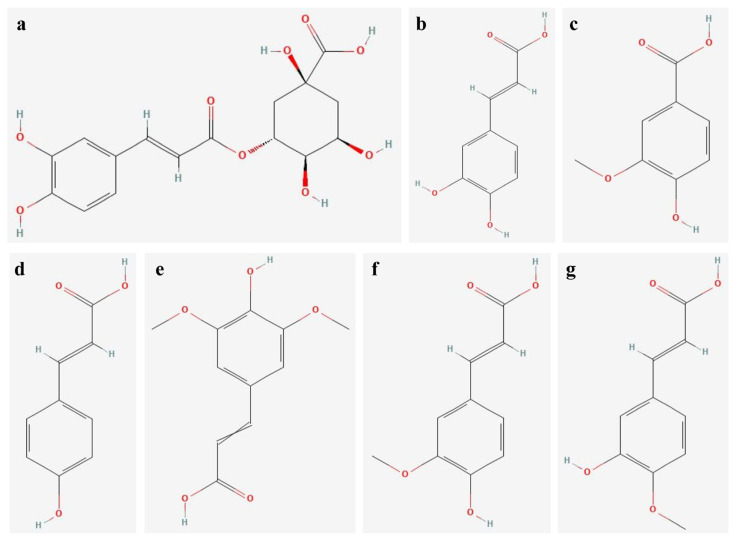
Chemical structures of phenolic acids identified in extracts of *L*. *aestivum* in vitro plants: (**a**) chlorogenic, (**b**) caffeic, (**c**) vanillic, (**d**) *p*-coumaric, (**e**) sinapic, (**f**) ferulic, (**g**) isoferulic.

Chlorogenic, caffeic, vanillic, *p*-coumaric, sinapic, ferulic and isoferulic acids were identified in *L*. *aestivum* plant cultures grown under different temperature conditions in this study (Table 3, Figure 4).

Limited information is available in the literature on the biosynthesis of individual phenolic acids in in vitro cultures of *L*. *aestivum* or other Amaryllidaceae plants. For instance, Morańska et al. [26] determined the content of eight phenolic acids (chlorogenic, *p*-hydroxybenzoic, caffeic, syringic, *p*-coumaric, ferulic, sinapic and benzoic acids) in *L*. *aestivum* plants exposed to LED light. By contrast, only three phenolic acids (vanillic, *p*-coumaric, ferulic) were identified in *L*. *aestivum* plants grown in natural habitats in Bulgaria [31].

The findings of this study indicate that in vitro cultures of *L*. *aestivum* may represent an important source of phenolic acids. It is worth emphasising that, to date, vanillic and isoferulic acids have not been identified in in vitro cultures of *L*. *aestivum*. However, these acids have various valuable pharmacological properties; for example, vanillic acid has antivenom, anti-inflammatory, antimicrobial, cardioprotective, hepatoprotective, free radical scavenging and antioxidant properties [62,63], while isoferulic acid has antioxidant properties, exhibits anti-inflammatory and antiapoptotic effects, and may play a role in controlling blood glucose levels [64]. It should be further noted that vanillic acid was present in the *L*. *aestivum* plants collected from the Black Sea region in Bulgaria [31].

In the present research, similar to the study by Morańska et al. [26], ferulic acid was found to dominate in *L*. *aestivum* plants. Additionally, a large amount of *p*-coumaric acid was found in the plants in this study (Table 3). It should be emphasised that ferulic acid exhibits strong antioxidant activity and is considered a promising molecule for the treatment of vascular disorders, while *p*-coumaric acid exhibits antioxidant and anticancer activities [65,66].

As shown in Table 3, the temperature used for the in vitro growth of *L*. *aestivum* plants influenced the production of phenolic acids. The stimulating effect of a temperature of 15 °C on the biosynthesis of six phenolic acids (i.e., chlorogenic, caffeic, *p*-coumaric, sinapic, ferulic and isoferulic acids) and of 30 °C on the biosynthesis of four phenolic acids’ (i.e., caffeic, vanillic, *p*-coumaric isoferulic acids) in in vitro cultures of *L*. *aestivum* was observed. Temperature’s effect on the biosynthesis of phenolic acids in Amaryllidaceae plants has not previously been studied. Still, prior research has shown that a temperature of 15 °C stimulates the production of the total phenolic acids in *Lavandula viridis* and of the phenolic compounds in *Ajuga bracteosa* in vitro plants [50,67]. Conversely, a high temperature or high heat stress influences the antioxidant systems of *Festuca trachyphylla* plants cultivated in pots and increases phenolic acid accumulation in such plants [68].

It should be underlined that the present observation concerning the highest phenolic acid content being recorded in *L*. *aestivum* in vitro plants grown at temperatures of 15 and 30 °C corresponded to the highest antioxidant enzyme activities noted under the same conditions (CAT: 15 °C and 30 °C, POD: 30 °C, SOD: 30 °C). To overcome stress constraints, plants adopt a number of alternative mechanisms that involve the synthesis of a wide range of specialized products, which serve as resistance tools. An antioxidative defence system and different metabolites, such as phenolic acids, help plants to survive under adverse conditions. It is also known that plants modify their phenolic metabolism in response to heat or cold stress to better tolerate unfavourable temperature conditions [69].

### 2.6. Effect of Temperature on Amaryllidaceae Alkaloid Biosynthesis

**Figure 5:**
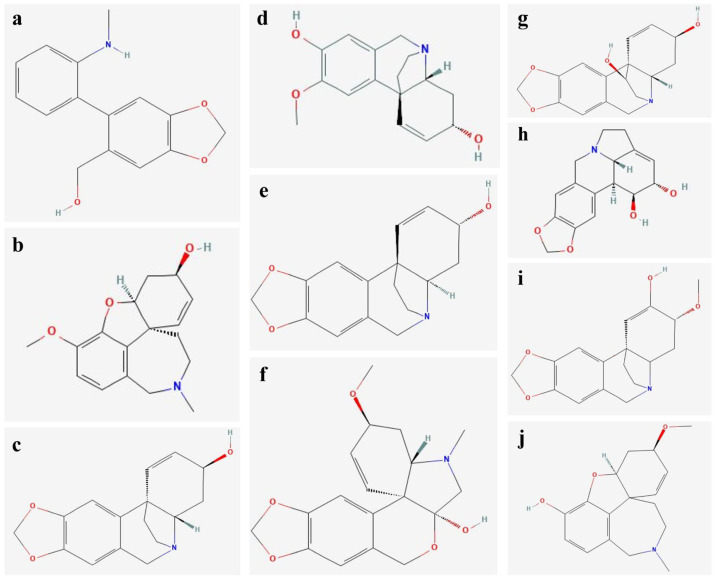
Chemical structures of Amaryllidaceae alkaloids identified in *L*. *aestivum* in vitro plants: (**a**) ismine, (**b**) galanthamine, (**c**) crinan-3-ol (vittatine), (**d**) demethylmaritidine, (**e**) crinine, (**f**) tazettine, (**g**) 11-hydroxyvittatine, (**h**) lycorine, (**i**) epiisohaemanthamine, (**j**) chlidanthine.

The qualitative analysis of the alkaloids obtained from *L*. *aestivum* plants under different temperature conditions was performed by means of gas chromatography–mass spectrometry (GC-MS). A total of 10 Amaryllidaceae alkaloids was identified from the different subgroups—that is: haemanthamine type (*para-para*): ismine [**1-a**], demethylmaritidine [**4-d**], 11-hydroxyvittatine [**7-g**], epiisohaemanthamine [**9-i**], crinine type (*para-para*): crinine [**5-e**], crinan-3-ol (vittatine) [**3-c**], tazettine type (*para-para*): tazettine [**6-f**]; lycorine type (*ortho-para*): lycorine [**8-h**], galanthamine type (*para-ortho*): galanthamine [**2-b**], chlidanthine [**10-j**] (Table 4, Figure 5).

Lycorine exhibited the highest percentage contribution in the alkaloid patterns, accounting for between 37.9% and 55.4% of the total ion chromatogram (TIC). The dominant contribution of crinine was also noted: 9.8–25.6% of the TIC (Table 4). It should be emphasised that lycorine is currently attracting significant research interest due to its strong anticancer activities against various types of cancer [5,6]. Studies have also shown that crinine is a very promising alkaloid due to its antiproliferative effects against human tumour cell lines [70].

The cultivation temperature of in vitro cultured *L*. *aestivum* plants has a significant impact on the biosynthesis of alkaloids. Here, the highest diversity of alkaloids (eight alkaloids) was observed in the plants cultured at 30 °C. Conversely, the lowest number of alkaloids (five) was noted in the plants grown at a temperature of 15 °C.

In the remaining temperature conditions, seven alkaloids were identified. It is worth emphasising, however, that only the extracts of plants grown at temperature of 15 °C showed the presence of ismine and tazettine. These alkaloids demonstrate strong biological and pharmacological activities. For instance, ismine has neuroprotective, antibacterial, antifungal and cytotoxic activities [71], while tazettine exhibits cytotoxic activities [70]. It should be further noted that ismine and tazettine are not typical alkaloids found in wild *L*. *aestivum* plants, which mainly contain galanthamine, epinorgalanthamine, narwedine, lycorine and ungiminorine [72]. Ismine and tazettine are also not typical alkaloids observed in in vitro cultures of *L*. *aestivum*. In fact, according to the literature, ismine has not previously been identified in any *L*. *aestivum* cultures [38], while tazettine was only noted in callus obtained on a medium containing 50 µM of picloram and plants grown on a medium enriched with glucose or fructose [24,73].

This study revealed a temperature of 20 °C to be optimal for the biosynthesis of galanthamine-type alkaloids: galanthamine and chlidanthine (16.2% and 2.8% of the TIC, respectively). The high percentage of the TIC of galanthamine was also noted for plants grown at 30 °C (10.2%). It should also be emphasised that the presence of chlidanthine was only recorded in plants grown at temperatures of 20 and 30 °C. While a temperature of 15 °C stimulated the biosynthesis of lycorine (55.4% of the TIC), a slightly lower percentage of the TIC of lycorine was noted in extracts obtained from plants cultivated at 25 and 30 °C (on average: 47.45% of the TIC).

The possibility of manipulating the biosynthesis of Amaryllidaceae alkaloids in *L*. *aestivum* shoot cultures cultivated in the Rita^®^ bioreactor based on the temperature was postulated by Ivanov et al. [38]. They observed temperatures of 18–22 °C to be favourable for galanthamine and 26 °C to be beneficial for lycorine biosynthesis in the shoots of *L*. *aestivum* plants, whereas a temperature of 18 °C had no significant influence on the galanthamine and lycorine percentages in the alkaloid mixture [38]. Interestingly, in the present study, a temperature of 15 °C stimulated lycorine and inhibited galanthamine biosynthesis in the *L*. *aestivum* plants. These results clearly indicate that *L*. *aestivum* plants respond to temperature stress differently than the shoots. Prior studies have shown that the effect of elicitation greatly depends on the stage of culture [74].

Quantitative analysis of the alkaloids showed the highest galanthamine content in the *L*. *aestivum* plants grown at 20 and 30 °C (107.38 and 104.09 µg/g DW, respectively) (Table 5).

These values were, on average, 18.2 times higher than the lowest value recorded for the plants cultured at 15 °C. Temperatures of 15 and 30 °C stimulated the biosynthesis of lycorine (content: 269.50 and 268.75 µg/g DW, respectively).

It should be noted that temperatures of 15 and 30 °C were the least favourable for *L*. *aestivum* plant growth, likely due to their response to temperature stress. At 15 °C, the lowest FW of the plants was noted, while at temperatures of 15 and 30 °C, the least leaves were formed. Moreover, the plants grown at 30 °C showed a tendency towards browning. In addition, the plants grown at temperatures of 15 and 30 °C were characterised by the highest CAT activity, while the highest POD and SOD activities were noted in the plants cultured at 30 °C. Furthermore, in the plants grown at temperatures of 15 °C and 30 °C, the highest content of phenolic acids was observed.

Plants can adapt to changes in abiotic stresses, such as temperature, by adjusting their metabolism. Generally, high/low temperature stress enhances specialized metabolite production in plants [75], which was also observed in this study in the case of galanthamine and lycorine biosynthesis in the *L*. *aestivum* plant cultures. These results contrast with the observations of Ivanov et al. [38] in shoot cultures of *L*. *aestivum*—namely, that the highest galanthamine yields coincided with the optimal growth conditions for biomass accumulation. This difference proves that the degree of organ differentiation and the culture conditions have a significant influence on galanthamine and lycorine biosynthesis. With regard to the influence of temperature on the biosynthesis of alkaloids under in vitro conditions, it has been shown that, for example, in plant cultures of *Papaver bracteautum* and *Papaver somniferum*, the biosynthesis of the morphine alkaloid was greatly influenced by the temperature, with optimal alkaloid production occurring at 18.5 and 20 °C [76]. In cell suspension cultures of *Catharanthus roseus*, the indole alkaloid content in the cells or in the medium was not affected by a change in temperature [77]. However, only limited data are available on the influence of temperature on the biosynthesis of alkaloids under in vitro conditions [78].

### 2.7. Effect of Temperature on Expression of Amaryllidaceae Alkaloid Biosynthesis Pathway Genes in L. aestivum In Vitro Plants

**Figure 6:**
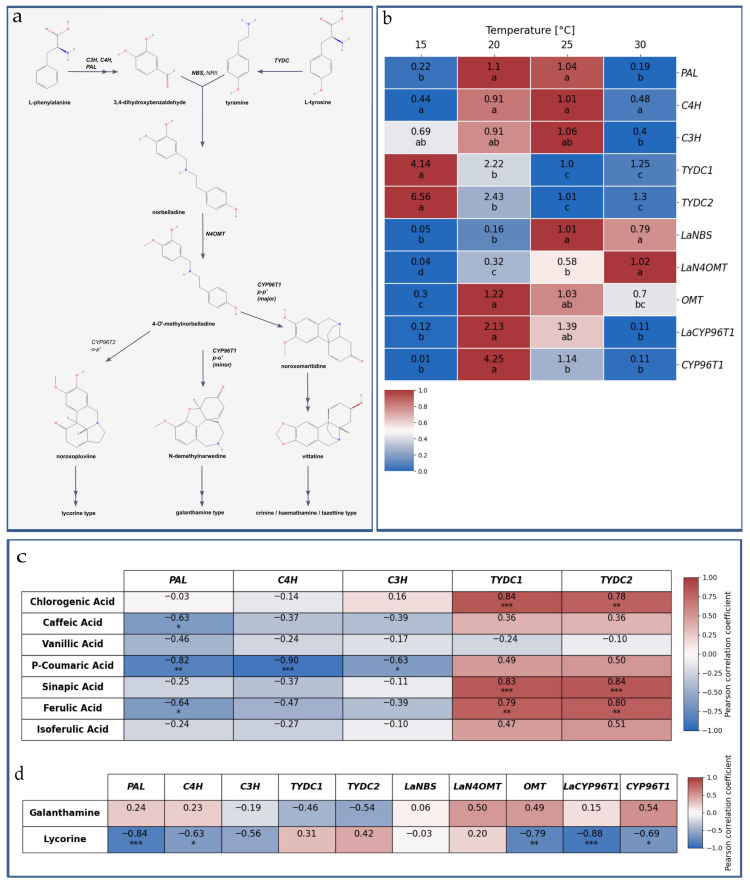
Genes engaged in the alkaloid biosynthesis pathway in *L*. *aestivum*. (**a**) The diagram illustrates proposed Amaryllidaceae alkaloids biosynthesis pathway. The genes analysed in presented research are written in bold font. (**b**) Heatmap of quantitative real-time PCR results of expression of ten Amaryllidaceae biosynthesis pathway genes in regard to the temperature conditions. The expression values were calculated using the comparative 2^−ΔΔCt^ method from three independent experiments. Actin was used as an internal reference. Different letters for expression values of each gene indicate a significance difference at *p* < 0.05 according to ANOVA and Duncan’s test. (**c**) Correlation coefficient between Amaryllidaceae alkaloid gene expression and phenolic acids as well as (**d**) galanthamine and lycorine content. Pearson correlation coefficient was calculated using scipy library in Python v.3.10 (*n* = 3, * *p* < 0.05, ** *p* < 0.01, *** *p* < 0.001 by Student’s *t*-test). Heatmap visualization was performed using the matplotlib and seaborn libraries in Python. Abbreviations of the studied genes: *PAL*—phenylalanine ammonia lyase; *C4H*—trans-cinnamate hydroxylase; *C3H*—p-coumarate hydroxylase; *TYDC*—tyramine decarboxylase; *NBS*—norbelladine synthase; *N4OMT*—norbelladine 4-*O*-methyltransferase; *CYP96T1*—cytochrome P450 monooxygenase 96T1.

This study analysed the expression of genes engaged in different steps of the Amaryllidaceae alkaloid biosynthesis pathway (Figure 6a). In terms of the temperatures tested, we have observed differences in the expression values of all the tested genes (Figure 6b). More specifically, the greatest differences were observed at 15 and 30 °C when compared with 20 and 25 °C. The most highly expressed gene at 15 °C, as well as the most highly expressed gene generally, was the *TYDC* gene that encodes the enzyme tyrosine decarboxylase (TYDC), which plays a critical role in the biosynthesis of tyramine and Amaryllidaceae alkaloids.

According to the literature, the expression of this gene is influenced by environmental factors, as part of the plant’s response mechanism. Tyramine is known to be one of the specialized metabolites that plays important antistress roles, and the accumulation of tyramine could be helpful for plants grown under stressful conditions [79,80]. It has previously been shown that two *TYDC* transcript variants are present in Amaryllidaceae plants [81,82]. Moreover, *TYDC1* may be involved in the synthesis of Amaryllidaceae alkaloids, whereas *TYDC2* may be responsible for the production of primary metabolites [83]. In this study, the transcript of *TYDC*2 was accumulated in higher amounts when compared with the transcript of *TYDC1.* However, it was clearly visible at 15 °C for both transcripts that the expression level decreased as the temperature increased.

Accumulation of the *TYDC* transcript was positively correlated with the chlorogenic, caffeic, *p*-coumaric, sinapic, ferulic and isoferulic acid contents (Figure 6c). It is worth noting that, when compared with the plants grown at 25 °C, which was an optimal temperature for *L*. *aestivum* growth in in vitro conditions (Table 1), in the plants grown at 15 °C, the accumulation of the *TYDC2* and *TYDC1* transcripts was about 6.5 and 4 times higher, respectively (Figure 6b).

It is also worth mentioning that, in addition to TYDC, the PAL enzyme plays a critical role in linking primary and secondary metabolism by transforming the amino acid phenylalanine into trans-cinnamic acid, which is also involved in the biosynthesis of several types of specialised metabolites. However, the expression of the *PAL* gene, as well as the *C4H* and *C3H* genes, which are involved in the phenylpropanoid pathway, displayed a similar expression pattern at all of the tested temperatures. The highest accumulations of these three transcripts were observed at 20 and 25 °C, while the lowest were seen at 15 and 30 °C. A similar expression pattern was observed for the *CYP96T1* transcripts (Figure 6b). Moreover, the *CYP96T1* transcripts at 15 and 30 °C were at the lowest levels among all of the tested genes and were inversely correlated with the lycorine content (Figure 6d). Yet this gene showed the highest expression at 20 °C, which favours galanthamine biosynthesis (Figure 6b). The *CYP96T1* gene encodes cytochrome P450 96T1 monooxygenase, which is the first enzyme known to exhibit phenol coupling activity, as characterised in monocots [84].

The specific reactions of the phenol coupling of 4′-*O*-methylnorbelladine (*para-ortho’*, *ortho-para’* and *para-para’*) lead to the formation of the main precursors of the different types of Amaryllidaceae alkaloids [83,84]. Recently, two transcripts of *CYP96T* were reported in *L*. *aestivum*, suggesting that LaCYP96T candidates could preferentially catalyse the *o-p*’ and *p-p*’ reactions but not the *p-o*’ reaction [85]. Taken together, the results available in the literature and those of the present study indicate that some other mechanisms are responsible for the lycorine content under temperature stress conditions. The lowest tested temperature had the strongest negative influence on *OMT* gene expression. In both transcript variants, the accumulation increased at higher temperatures (Figure 6b). It is known that N4OMT is responsible for the methylation of norbelladine, the compound from which all Amaryllidaceae alkaloids are derived [81,86].

When referring to the gene expression profile of the Amaryllidaceae alkaloid biosynthesis pathway and the content of galanthamine and lycorine, it may appear that the extreme tested temperatures—namely, 15 and 30 °C—had an influence on the Amaryllidaceae alkaloid precursors’ biosynthesis and on the expression of the Amaryllidaceae alkaloids’ biosynthesis-specific genes and the lycorine content. At 30 °C, the highest accumulation of galanthamine was recorded, although lycorine was the predominant Amaryllidaceae alkaloid. The correlation analysis for galanthamine revealed no significant relation (Figure 6d).

## 3. Materials and Methods

### 3.1. In Vitro Experimental Cultures

*Leucojum aestivum* in vitro plants obtained by somatic embryogenesis according to the procedure described by Ptak et al. [22,73] were used in this study. Five properly formed plants (average height 2 cm, weight 0.2–0.5 g) were placed in jars in solid Murashige and Skoog medium (MS, [87]) enriched with 5 µM of zeatin (Sigma-Aldrich, St. Louis, MO, USA). The pH was adjusted to 5.8. For each combination, a total of 175 plants were cultured; for the overall study, a total of 700 plants were used. The cultures were grown for four weeks in a climatic chamber (Adaptis-A1000TC, Conviron, Winnipeg, MB, Canada) at different temperatures—namely, 15, 20, 25 and 30 °C, with 25 °C used as the control. Moreover, 70% relative humidity and a white fluorescent lamp (390–760 nm, OSRAM Fluora 36W/77, Munich, Germany) (with a 16/8 h photoperiod [day/night], PPFD at 60 μmol m^−2^ s^−1^) were applied in a climatic chamber. After four weeks of culture, the numbers of leaves and roots developed from one plant were counted, and the increase in the FW (final fresh mass–initial fresh mass) of the plants was determined. The DW was determined after the plants had been freeze-dried for 72 h. The DW content of the plant tissue was calculated according to the following formula:

DW [%] = DWL × 100/FWF

where DWL is the dry weight after lyophilisation and FWF is the final FW. The lyophilised and powdered samples were stored at −80 °C until needed for further analysis.

### 3.2. Determination of Photosynthetic Pigments

Using a microplate reader (Synergy II; BioTek, Winooski, VT, USA), spectrophotometric analysis was performed to ascertain the amount of photosynthetic pigments present in the plant tissue. Plant material weighing 5 mg was extracted using 80% ethanol. After 15 min of shaking, the samples were centrifuged (20,000× *g*). The wavelengths used for the spectrophotometric measurement were as follows: 470, 648 and 664 nm. The total amount of carotenoids and the content of chlorophyll *a* and *b* were determined using Lichtenthaler and Wellburn’s [88] formula:

Chl. a = 13.36 A664 − 5.19 A648

Chl. b = 27.43 A648 − 8.12 A664

Car = (1000 A470 − 2.13 Chl. a − 97.64 Chl. b)/209

where A is the absorbance value for the wavelength, Chl. a is the concentration of chlorophyll *a*, Chl. b is the concentration of chlorophyll *b* and Car is the concentration of total carotenoids.

### 3.3. Determination of Soluble Sugars

In accordance with the method reported by Dubois et al. [89], the amount of soluble sugars was estimated spectrophotometrically using the phenol–sulfuric acid method. Ethanol (80%) was used to extract 5 mg of plant material, while 150 μL of distilled water, 50 μL of the ethanolic plant extract, 200 μL of 5% phenol solution and 1 mL of concentrated H_2_SO_4_ made up the reaction mixture. A Bio-Tek Synergy II microplate reader was used to measure the absorbance at 490 nm.

### 3.4. Antioxidant Enzyme Activity Analysis

Plant material (100 mg) was homogenised at 4 °C with 0.05 M phosphate buffer (pH 7.0) containing 0.1 mM EDTA. The activities of the tested antioxidant enzymes—namely, SOD, CAT and POD—were measured spectrophotometrically (Synergy II; Bio-Tek, Winooski, VT, USA). The SOD activity was measured according to the cytochrome method [90] at λ = 550 nm. The CAT activity was assessed by measuring the rate of H_2_O_2_ decomposition using the method reported by Aebi [91] at λ = 240 nm. The POD activity was determined by measuring the amount of oxidation products of 1% *p*-phenylenediamine in the presence of H_2_O_2_ at λ = 485 nm [92]. The enzymatic activity was expressed in relation to the total quantity of proteins found in the shoots. This total protein content was calculated using Bradford’s technique [93].

### 3.5. Phenolic Acid Analysis

Lyophilised and powdered plants (approximately 5 mg) were extracted twice with 100 µL ethanol/water (1:1) and 0.01% HCl (Chempur, Piekary Śląskie, Poland). During the extraction, the samples were sonicated (10 min) and centrifuged for 10 min at 15,000 rpm. The supernatants were combined. The analyses were carried out using the HPLC system (Agilent Technologies, Santa Clara, CA, USA), which consists of an ACQUITY BEHC18 1.7 μm 2.1 × 100 mm analytical column. The solvents used were water with 0.1% formic acid (A) and acetonitrile with 0.1% formic acid (B) (Merck SA, Darmstadt, Germany). A gradient was set from 15% to 90% B in 8 min. The HPLC system used was an Agilent Technologies 1260 with a binary pump and a QQQ 6410 mass spectrometer as a detector, and 2 µL of extract was injected into the HPLC system.

Characteristic multiple reaction mode (MRM) fragmentation ions were used for the identification and quantitation of the phenolic acids: chlorogenic, caffeic, vanillic, *p*-coumaric, sinapic, ferulic and isoferulic. Commercially available standards were used to prepare the calibration curves (Sigma-Aldrich, St. Louis, MO, USA). The contents of the different phenolic acids in the raw material were calculated against calibration curves plotted as the dependence of the surface area under the peaks for the standard phenolic acids.

### 3.6. Amaryllidaceae Alkaloid Analyses

The alkaloids were extracted from lyophilised plants (150 mg) and then purified and analysed using a GC-MS system comprising QP2010-Shimadzu equipment (Shimadzu, Kyoto, Japan) operating as described by Saliba et al. [94].

The identification of the alkaloids was performed by comparing the measured data with those of the authentic compounds (galanthamine and lycorine), or with previous data [95]. The alkaloids were quantified using LC-MS equipment constituted by UPLC advance and EVOQ Elite (Bruker Daltonics, Bruker, Bremen, Germany). An internal standard calibration method, along with a nine-point calibration curve (R^2^ = 0.99) using authentic galanthamine and lycorine (Sigma-Aldrich, St. Quentin Fallavier, France), was used for quantitative analysis of alkaloids.

### 3.7. Gene Expression Analysis

The total RNA was isolated using a Total RNA Mini Kit (A&A Biotechnology, Gdańsk, Poland) from approximately 100 mg of frozen plant material that was completely ground in liquid nitrogen. The isolation procedure was performed according to the manufacturer’s instructions. The quality and quantity of the RNA were checked spectrophotometrically (NanoDrop 2000c; Thermo Fisher Scientific, Waltham, MA, USA), while the integrity was verified by performing 1.5% (*w*/*v*) denaturing agarose gel electrophoresis. For the reverse transcription (Maxima First Strand cDNA Synthesis Kit for RT-qPCR with dsDNase; Thermo Fisher Scientific), 1 μg of RNA was used with the values of 260/230 higher than 1.8 and 280/260 nm absorption ratio 1.8–2.2. The cDNA was diluted 25-fold using nuclease-free water (Sigma-Aldrich, St. Louis, MO, USA) for the qRT-PCR (7500 Fast Real-Time PCR System; Applied Biosystems, Waltham, MA, USA). The reaction mix contained 2.5 μL of diluted cDNA, 5.0 μL of 2×Power Up Sybr Green Master Mix, 0.5 μL of each primer (Table 6) and ddH_2_O in a final volume of 10 μL.

Three biological replicates for each sample and three technical replicates for each biological replicate with a no-template control were used. The real-time PCR conditions for the amplification were 95 °C for 10 min, followed by 95 °C for 15 s, with an annealing temperature 60 °C for 1 min for 40 cycles. In order to verify the specificity of the primers, a melting curve was also analysed. The actin gene was used as an internal reference, and the relative transcript level was normalised for each sample to that of 25 °C using the 2^−ΔΔCT^ method [96].

**Table 6:** Primers used for gene expression analysis.

Gene	Primer Sequence Forward/Revers (F/R) (5′–3′)	Source and Species
*PAL*	F: CAAAGTGCAGAGCAACATAATCAAG	[58] *Lycoris longituba*
	R: TTCACTGTGCTCTTCAAATTCTCC	
*C4H*	F: GTCAGAGGAATCTCGTAGTCGTGTC	[58] *Lycoris longituba*
	R: CTCACCGTACACTGTAAAGACCATG	
*C3H*	F: CAGGTGCTTCGCCGAGTGG	[58] *Lycoris longituba*
	R: CCTCACCTTCACGTAGTGGG	
*TYDC1*	F: TGGTTTTAATATTGTGGGTTTCAAT	[97] *Narcissus pseudonarcissus*
	R: TTCACTAGCTGTGCCTTGAATTACT	
*TYDC2*	F: GTAATTCAAGGCACAGCTAGTGAAG	[97] *Narcissus pseudonarcissus*
	R: ATAAACCACAAGCTTTTCAAGTGAT	
*LaNBS*	F: AACGGGATCCATGAAGGGAAGTCTCTCCCATGAG	[82] *Leucojum aestivum*
	R: ACGCAAGCTTCTACGCTACAATAGCTTTTTGCTCC	
*LaN4OMT*	F: GGTGCTAGCCAAGATGATTA	NCBI: MW971978/ *Leucojum aestivum*
	R: CGTCGACAAATAGTCACTCC	
*OMT*	F: AAGCTTGTCAGGGTTGGAGG	[58] *Lycoris longituba*
	R: TACACTCCTCCTCTTCCGGA	
*LaCYP96T1*	F: GCTCCGCTAGATCTTCAAGC	NCBI: MW971979/ *Leucojum aestivum*
	R: AGCTTTCGCGAATGGTACGG	
*CYP96T1*	F: TGCTATGGCGAGGATGAAGG	[58] *Lycoris longituba*
	R: ACATGTCCCTTCACCATCTG	
*Actin*	F: GATAGAACCTCCAATCCAAACACTA	[97] *Narcissus pseudonarcissus*
	R: GTGTGATGTGGATATTAGGAAGGAC	

Abbreviations of the studied genes: *PAL*—phenylalanine ammonia lyase; *C4H*—trans-cinnamate hydroxylase; *C3H*—p-coumarate hydroxylase; *TYDC*—tyramine decarboxylase; *NBS*—norbelladine synthase; *N4OMT*—norbelladine 4-*O*-methyltransferase; *CYP96T1*—cytochrome P450 monooxygenase 96T1.

### 3.8. Statistical Analysis

The results are expressed as mean values ± standard errors (SE). Statistical analysis of the experimental data was performed by means of an analysis of variance. The differences between the means were determined using Duncan’s multiple range test at *p* < 0.05.

## 4. Conclusions

To our knowledge, this study provides the first comprehensive assessment of the effect of temperature on Amaryllidaceae alkaloid and phenolic acid biosynthesis in *Leucojum aestivum* plant cultures. A temperature of 30 °C was associated with increased alkaloid biosynthesis. At this temperature, the highest diversity of Amaryllidaceae alkaloids and the highest galanthamine and lycorine contents were recorded. Additionally, the biosynthesis of galanthamine was stimulated at 20 °C and that of lycorine at 15 °C. *CYP96T1* expression peaked at 20 °C and showed a strong negative correlation with lycorine content, suggesting temperature-dependent modulation of the metabolic flux among Amaryllidaceae alkaloid pathways rather than a direct regulatory relationship. Regarding phenolic acids, the positive effect of a temperature of 15 °C was observed, as was the highest expression of the *TYDC* gene. Overall, these findings indicate that temperature acts as an effective elicitor influencing the biosynthesis of key specialized metabolites in *L. aestivum* plant cultures, and that it can be exploited to optimize metabolite production in biotechnological systems.

This study also sheds additional light on the not fully understood metabolic pathway of Amaryllidaceae alkaloids biosynthesis and suggests that the molecular mechanism of their biosynthesis may be regulated by temperature.

## Figures and Tables

**Table 1:** Effect of temperature on morphological properties of *L*. *aestivum* plants.

Temperature [°C]	Number of Leaves per Plant	Number of Roots per Plant	FW Increments of Plants [mg]	DW Accumulation in Plants [%]
15	1.24 ± 0.09 ^c^	1.40 ± 0.20 ^a^	60 ± 0.03 ^c^	24.94 ± 3.10 ^b^
20	2.32 ± 0.11 ^b^	0.52 ± 0.10 ^b^	160 ± 0.04 ^b^	25.98 ± 2.16 ^b^
25	2.68 ± 0.10 ^a^	1.28 ± 0.12 ^a^	430 ± 0.03 ^a^	43.19 ± 0.02 ^a^
30	1.24 ± 0.09 ^c^	0 ± 0.00 ^c^	170 ± 0.49 ^b^	25.22 ± 0.03 ^b^

Values are expressed as mean ± SE (*n* = 35). Different letters indicate a significance difference at *p* < 0.05 according to ANOVA and Duncan’s test. FW: fresh weight, DW: dry weight.

**Table 2:** Effect of temperature on photosynthetic pigment (chlorophyll *a*, *b*, carotenoids) concentrations in extracts of *L*. *aestivum* in vitro plants.

Temperature [°C]	Chlorophyll *a* [µg/g FW]	Chlorophyll *b* [µg/g FW]	Carotenoids [µg/g FW]
15	38.76 ± 4.60 ^a^	26.35 ± 2.46 ^a^	13.06 ± 2.22 ^a^
20	44.33 ± 4.88 ^a^	27.29 ± 2.82 ^a^	12.55 ± 1.80 ^a^
25	38.10 ± 4.53 ^a^	26.88 ± 2.73 ^a^	9.86 ± 1.24 ^a^
30	31.19 ± 2.80 ^a^	21.08 ± 1.97 ^a^	8.46 ± 1.73 ^a^

Values are expressed as mean ± SE (*n* = 5). Different letters indicate a significance difference at *p* < 0.05 according to ANOVA and Duncan’s test. FW: fresh weight.

**Table 3:** Effect of temperature on phenolic acid concentrations in extracts of *L*. *aestivum* in vitro cultures.

Phenolic Acids [µg/g DW]	Temperature [°C]			
	15	20	25	30
chlorogenic	0.19 ± 0.003 ^a^	0.16 ± 0.013 ^b^	0.13 ± 0.001 ^c^	0.10 ± 0.005 ^d^
caffeic	1.40 ± 0.147 ^a^	0.95 ± 0.086 ^b^	1.05 ± 0.004 ^b^	1.36 ± 0.065 ^a^
vanillic	2.26 ± 0.018 ^b^	1.24 ± 0.039 ^c^	2.53 ± 0.120 ^a^	2.54 ± 0.030 ^a^
*p*-coumaric	3.44 ± 0.094 ^a^	2.83 ± 0.214 ^b^	2.68 ± 0.131 ^b^	3.20 ± 0.192 ^ab^
sinapic	0.75 ± 0.013 ^a^	0.58 ± 0.069 ^b^	0.50 ± 0.041 ^b^	0.45 ± 0.033 ^b^
ferulic	4.69 ± 0.257 ^a^	2.68 ± 0.160 ^c^	2.62 ± 0.196 ^c^	3.39 ± 0.210 ^b^
isoferulic	0.58 ± 0.115 ^a^	0.48 ± 0.072 ^a^	0.38 ± 0.068 ^a^	0.47 ± 0.068 ^a^

Values are expressed as mean ± SE (*n* = 3). Different letters indicate a significance difference at *p* < 0.05 according to ANOVA and Duncan’s test. DW: dry weight.

**Table 4:** Amaryllidaceae alkaloids identified by GC-MS (% of TIC) in *L. aestivum* in vitro plants grown in different temperature conditions. TIC: Total Ion Chromatogram, (-): no alkaloid.

No	Alkaloid	Formula	Retention Time [min]	Base Peak	Temperature [°C]			
					15	20	25	30
1	Ismine	C_15_H_15_NO_3_	13.37	238	0.4	-	-	-
2	Galanthamine	C_17_H_21_NO_3_	15.02	286	-	16.2	9.1	10.2
3	Crinan-3-ol (vittatine)	C_16_H_19_NO_3_	15.57	272	-	10.1	0.9	0.7
4	Demethylmaritidine	C_16_H_19_NO_3_	16.48	201	9.9	7.5	3.6	7
5	Crinine	C_16_H_17_NO_3_	16.85	239	23.6	11.7	9.8	25.6
6	Tazettine	C_18_H_21_NO_5_	18.50	246	3	-	-	-
7	11-Hydroxyvittatine	C_16_H_17_NO_4_	19.27	258	-	13.1	13	10.1
8	Lycorine	C_16_H_17_NO_4_	19.80	226	55.4	37.9	50.2	44.7
9	Epiisohaemanthamine	C_17_H_19_NO_4_	20.51	301	-	-	2.2	2.9
10	Chlidanthine	C_17_H_21_NO_3_	20.77	228	-	2.8	-	1

**Table 5:** Effect of temperature on galanthamine and lycorine contents in extracts of *L*. *aestivum* in vitro plants.

Temperature [°C]	Galanthamine [µg/g DW]	Lycorine [µg/g DW]
15	5.81 ± 0.001 ^b^	269.50 ± 0.013 ^a^
20	107.38 ± 0.02 ^a^	178.53 ± 0.006 ^b^
25	35.27 ± 0.016 ^b^	209.43 ± 0.016 ^b^
30	104.09 ± 0.012 ^a^	268.75 ± 0.002 ^a^

Values are expressed as mean ± SE (*n* = 3). Different letters indicate a significance difference at *p* < 0.05 according to ANOVA and Duncan’s test. DW: dry weight.

## Data Availability

All data are included in this article.
